# Feature-Specific Encoding Flexibility in Visual Working Memory

**DOI:** 10.1371/journal.pone.0050962

**Published:** 2012-12-28

**Authors:** Aki Kondo, Jun Saiki

**Affiliations:** 1 Research Center for Advanced Science and Technology, The University of Tokyo, Tokyo, Japan; 2 Graduate School of Human and Environmental Studies, Kyoto University, Kyoto, Japan; CSIC-Univ Miguel Hernandez, Spain

## Abstract

The current study examined selective encoding in visual working memory by systematically investigating interference from task-irrelevant features. The stimuli were objects defined by three features (color, shape, and location), and during a delay period, any of the features could switch between two objects. Additionally, single- and whole-probe trials were randomized within experimental blocks to investigate effects of memory retrieval. A series of relevant-feature switch detection tasks, where one feature was task-irrelevant, showed that interference from the task-irrelevant feature was only observed in the color-shape task, suggesting that color and shape information could be successfully filtered out, but location information could not, even when location was a task-irrelevant feature. Therefore, although location information is added to object representations independent of task demands in a relatively automatic manner, other features (e.g., color, shape) can be flexibly added to object representations.

## Introduction

We can effortlessly and simultaneously perceive objects that consist of many features such as color, shape, and location. However, these features are well known to be processed in separate brain regions. This discrepancy between distinct neural processing of individual features and the perception of an integrated single object is known as “the binding problem”. The binding problem has primarily been studied in the context of visual perception [1], [2]. Then, how are object representations with integrated features stored in visual working memory (VWM)?

Luck and Vogel [3] and, more extensively, Vogel, Woodman and Luck [4], showed that multiple features of a single object could be bound together as a single unit in VWM. They showed that an estimated capacity for multi-feature objects was as good as the capacity for each simple feature about 3–5 objects in a short-term change-detection task. This basic finding has been replicated repeatedly, and it seems that VWM stores features in an integrated way. However, it remains unclear how object features are bound together in VWM. In the present study, we investigated the nature of feature bindings in VWM by examining (1a) how flexible object features could be bound in VWM. Especially, we focused on whether we can selectively bind only task relevant visual features, or all the visual features are automatically bound independent of task relevancy. (1b) All of the visual features are bound in the same way, or a specific feature plays a special role in binding. (2) Based on the previous study by Wheeler and Treisman [5], in which they reported that accuracy in VWM binding task depends on task demands (especially retrieval difficulty), we are interested in whether specific feature binding can be easily retrieved than other feature bindings.

### (1a, b) Feature Binding in Visual Working Memory

Related to our question (1a), Treisman and Zhang [6] investigated interference from task-irrelevant features in change detection tasks, and showed that changes in feature binding led to significant interference. This suggests that binding occurs automatically when attention is directed to an object during encoding. They also showed that in a binding change detection task, location changes produced significant interference, suggesting that feature bindings are retrieved through their locations.

In the present study, we further focused on whether all features of attended objects are always bound, or selective feature could be bound depending on the task demand. We consider two possibilities. First, as suggested by Treisman and Zhang [6], it may be that all object features are always bound to form an object representation in VWM. In this case, even the task-irrelevant features would be automatically encoded and maintained in VWM, leading to interference. Second, it may be that only task-relevant features are bound selectively and flexibly. In this case, the irrelevant features would not interfere with memory for relevant features.

About the question (1b), we hypothesized that specific feature could play a special role in VWM binding. This idea is based on the object file theory introduced by Kahneman, Treisman, & Gibbs [7]. Object files are episodic visual representations that use spatiotemporal information to track entities over time and motion, and store (and update) information about these representations. Given the special role of location information in object files, when location is irrelevant, it cannot be filtered out, unlike other features such as color and shape.

In the present study, we evaluated the flexibility of object representations in VWM, using a task where participants were asked to detect whether specific features changed (called “relevant-feature switch detection task” hereafter), instead of a conventional change detection task. In the relevant-feature switch detection task, participants were instructed to monitor the conjunction of color-shape, color-location, or shape-location, and were required to judge whether the stimulus sequence included a switch type on the specified conjunction of two features, while ignoring changes to the other feature. If we can create object representations that only combine the task-relevant features (we call this the partial object file theory), there will be no difference in performance between the color-shape, color-location, and shape-location conditions. On the other hand, if we always create object representations that combine all presented object features (we call this the full object file theory), then strong interference will be observed in all conditions. If encoding flexibility is feature-specific (we call this the feature-specific theory), then according to object file theory, strong interference will only be observed in the color-shape condition.

### (2) Feature specific retrieval difficulty in Working Memory binding

As the second main question, we are interested in whether specific feature binding can be remembered easier than other feature bindings. Wheeler and Treisman [5] compared a single-probe condition with a whole-probe condition, in which only one object was presented in the probe display in the single-probe condition whereas the whole-probe display needed to be compared with the initial display in the whole probe condition. They found that performance in the binding condition was significantly better in the single-probe compared to the whole-probe condition. This single-probe advantage was interpreted as a reduction of interference and/or facilitation of memory retrieval by the single-probe.

Based on their result, the present study further focused on the question whether single probe advantage in the retrieval stage would occur in all combinations of feature binding or the advantage would be found in a specific feature binding. Here we systematically investigate the single-probe advantage in a variety of different conditions using the same stimulus set. In terms of the single-probe advantage, both partial and full object file theories described above predict a comparable advantage across conditions, because they assume comparable retrieval costs and interference. The feature-specific theory predicts a larger single-probe advantage in the color-shape condition, due to greater interference from location information.

## Experiment 1A: Simple Change Detection Task

In Experiment 1A, we conducted a simple switch detection task, using stimuli defined by a conjunction of three features (color, shape, and location). We used this condition as a baseline because all features were relevant to the task, so there could be no filtering cost for irrelevant features.

### Methods

#### Participants

Fifteen undergraduate students at Kyoto University received class credit for their participation in this study. All participants had normal color vision and normal or corrected-to-normal visual acuity. The procedures were approved by the internal review board of Graduate School of Human and Environmental Studies, Kyoto University, and written informed consent was obtained from all participants prior to the testing.

#### Materials

Experiments were run on a standard PC with a VSG2/5 graphics card (Cambridge Research Systems, Ltd., Kent, UK). Stimulus presentation was controlled with Matlab (Mathworks®, Cambridge, UK). Stimuli were presented on a 21-inch CRT monitor (FlexScan-F980, Eizo Nanao Corporation, JP) at a viewing distance of approximately 70 cm. Responses were collected using a button box (CT3, Cambridge Research Systems, Ltd., Kent, UK).

The stimuli were simple objects defined by color, shape, and spatial location, and subtended approximately 0.75° of visual angle. Objects were six shapes (circle, triangle, square, cross, star, and pillar) presented in one of seven colors (red, yellow, green, blue, violet, white, and black). These colors were selected for use on the basis of ease of discriminability. Items were presented at randomly selected locations within an invisible 3×3 matrix (1.38°×1.38°), except for the center location. The objects presented on each trial were selected at random, with the constraint that no feature was repeated within a single trial.

**Figure 1:**
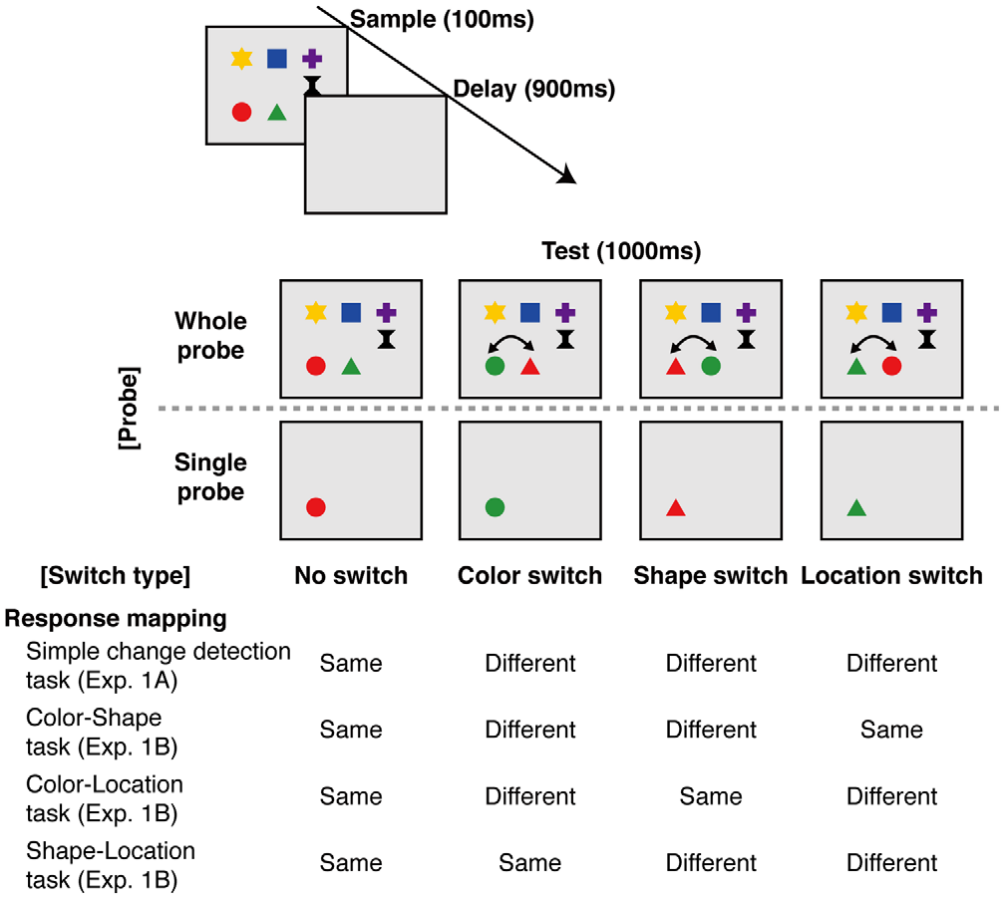
Schematic illustration of the relevant-feature switch detection task. Four switch types (no-change, color-switch, shape-switch, and location-switch) and response mappings in Experiments 1a and 1b are illustrated.

Examples of the sample and test displays are illustrated in Figure 1. In this study, participants were detecting feature switches between two objects, such that the pairing of features changed, but the feature identities did not. A total of four switch types were possible: no switch, color switch, shape switch, and location switch (Figure 1). For example, a sample display containing a red circle located at coordinate (xa, ya) and a green triangle located at (xb, yb) might be followed with a test display containing a green circle at (xa, ya) and a red triangle at (xb, yb) in the color-switch condition, a red triangle at (xa, ya) and a green circle at (xb, yb) in the shape-switch condition, or a red circle at (xb, yb) and a green triangle at (xa, ya) in the location switch condition. Note that detection of a color-switch requires memory for the conjunction of each object's color and spatial location, whereas detection of color-replacement does not. Performance on each feature-switch condition is the critical measure of memory for the conjunction of two features in this paradigm.

#### Procedure

Each experimental trial began when the participant initiated a key press. In the first display, six random digits (e.g., “719632”) were presented for 2000 ms, and participants were instructed to rehearse the digits aloud throughout the trial to minimize the use of verbal working memory [4]. After the digits disappeared, the sample display consisting of six objects was presented for 100 ms. Following a blank interval of 900 ms, the test display was presented for 2000 ms. During the delay period, features of two objects, either of color, shape or location, switched on 50% of the trials. In the whole-probe condition, all objects were presented at test, whereas in the single-probe condition, only one object was shown. Participants made a same or different response using a response box, pressing a left button for same, and a right button for different. Accuracy was emphasized rather than speed.

There were two experimental blocks, each containing 96 trials. A rest was provided between blocks. Within each block, switch type (no-switch, color-switch, shape-switch, and location-switch) and probe condition (whole-probe and single-probe) were randomized. In each probe condition, there were 24 trials for each switch type, resulting in a total of 192 experimental trials. Participants received 32 practice trials at the beginning of each block.

### Results and Discussion

**Figure 2:**
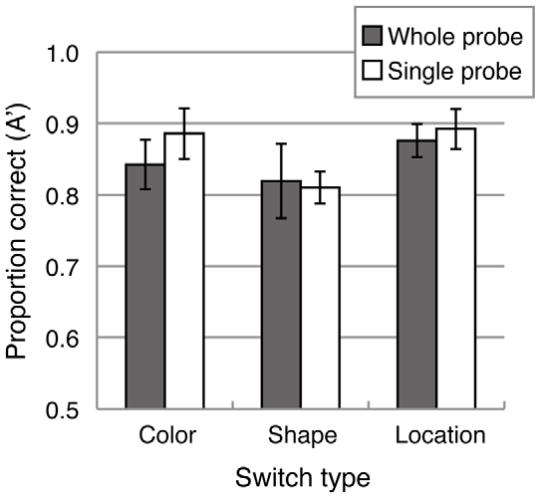
Mean sensitivity (A') for each switch type and probe condition in Experiment 1A.

Figure 2 shows the mean of a nonparametric index of sensitivity (A') [8] for each switch type and probe condition. A 3 (switch type: color, shape and location) ×2 (probe: whole-probe and single-probe) ANOVA revealed a significant main effect of switch type (F(2, 28)  = 15.787, p<.001), but no significant main effect of probe condition (F(1, 14)  = 0.094, n.s.) or interaction (F(2, 28)  = 2.196, n.s.). Multiple comparisons (Ryan's method) showed that mean sensitivity (A') in the color-switch and location-switch conditions were significantly higher than the shape-switch condition (p<0.05). Although the overall level of task performance in this three-feature conjunction task (average accuracy of 85%) is comparable to the previous study that used conjunctions of two features [5], the single-probe advantage was not observed.

## Experiment 1B: Relevant-Feature Switch Detection Task

In Experiment 1B, a relevant-feature switch detection task was used. On each trial, participants were instructed to monitor for either color-shape, color-location, or shape-location conjunctions, and to judge whether the stimulus sequence included a switch on the pre-specified feature dimensions. Notably, this task only had two response alternatives, like a simple change-detection task, but required distinguishing between color-, shape- and location-switch types, as described above. Therefore, if the single-probe advantage observed in Wheeler and Treisman [5] also occurs for partial object files, then we would expect facilitation for single-probes.

### Methods

#### Participants

Forty-five undergraduate students at Kyoto University received class credit for participation in this study. All participants had normal color vision and normal or corrected-to-normal visual acuity. The procedures were approved by the internal review board of Graduate School of Human and Environmental Studies, Kyoto University, and written informed consent was obtained from all participants prior to the testing. Participants were randomly assigned to one of three switch-detection tasks: color-shape switch, color-location switch, or shape-location switch.

#### Procedure

The task was to detect a switch on pre-specified features. For example, in the color-shape switch detection task, participants were required to respond “different” if there was a color-switch or shape-switch, and “same” if there was no-switch or a location-switch (Figure 1). Thus, although the task has two response alternatives like a simple change-detection task, participants needed to monitor the relevant features to make a correct response. Each trial began with the appearance of six digits (2000 ms). Participants were instructed to rehearse the digits aloud throughout the trial. Then, a display consisting of six objects appeared briefly (100 ms), followed by a blank interval (900 ms), and then a display consisting of one (single-probe condition) or six (whole-probe condition) objects was presented (2000 ms). Participants were instructed to judge whether the conjunctions of color and shape (or color and location, shape and location) were the same or different between the two displays, and to ignore changes in location (or shape, color). Participants pressed the left button for “same” and the right button for “different.” Accuracy was emphasized over speed.

There were two experimental blocks of 96 trials for a total of 192 trials. Within each block, switch type and probe conditions were randomized. Participants were given 32 practice trials before each experimental block.

### Results and Discussion

**Figure 3:**
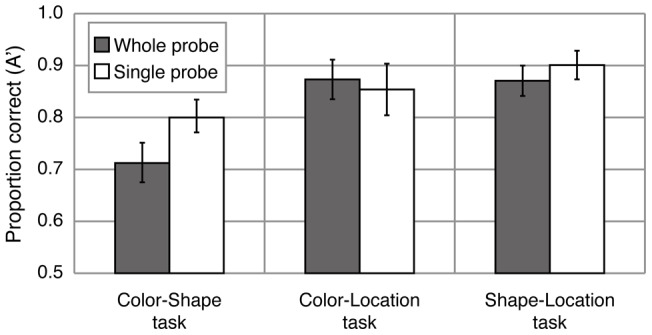
Mean sensitivity (A') for each task and probe condition in Experiment 1B.

Figure 3 shows mean A' for each task and probe condition. A 3×2 mixed ANOVA with task (color-shape, color-location and shape-location) as a between-subjects factor and probe condition (whole-probe and single-probe) as a within-subjects factor revealed a significant main effect of task (F(2, 42)  = 23.404, p<.0001), probe condition (F(1, 14)  = 5.737, p<.05) and a significant interaction (F(2, 42)  = 4.778, p<.05). The analysis of the simple main effect revealed a significant single-probe advantage on change detection for color-shape task (F(1, 42)  = 13.202, p<.001), but not for shape-location (F(1, 42)  = 0.536, n.s.) or color-location (F(1, 42)  = 1.556, n.s.) task. Therefore, the single-probe advantage largely depended on the features to be bound.

**Figure 4:**
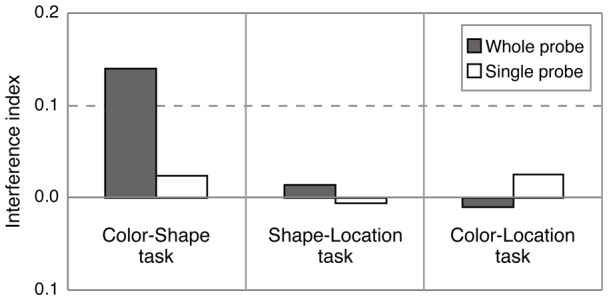
Mean interference indices for each task and probe condition in Experiment 1B.

The effect of task-irrelevant features may depend on the task-relevant feature conjunctions (color-shape, color-location, and shape-location) and/or probe-type (whole-probe and single-probe). To quantify the interference from the task-irrelevant feature, we computed an interference index for each condition (Figure 4). Interference indices (i) were obtained by subtracting the false alarm rate when no change occurred from that when the irrelevant feature changed. The interference index is positive if the task-irrelevant feature interferes with detecting a change on the task-relevant feature, and zero if there is no interference from the task-irrelevant feature. A 3 (task: color-shape, color-location, and shape-location) ×2 (probe type: whole-probe and single-probe) ANOVA only revealed a significant interaction between switch type and probe condition (F(2, 42)  = 3.205, p = .05). The analysis of the simple main effect revealed a significant difference in the interference index between whole-probe and single-probe for color-shape task, (F(1, 42)  = 13.202, p<.001), but not for color-location (F(1, 42)  = 1.556, n.s.) and shape-location (F(1, 42)  = 0.536, n.s.) task. These results suggest that the single-probe advantage observed in the color-shape task can be attributed to differences in interference from changes to the irrelevant feature (spatial location) between whole-probe and single-probe conditions. To evaluate whether significant interference was observed in each condition, the interference indices were compared with zero (no interference). The interference index for the whole-probe color-shape switch condition was significantly higher than zero (p<.005), whereas the other five conditions were not significantly different from zero (ps >.34).

The results of Experiment 1B support the predictions of object file theory. Contrary to expectation, the single-probe advantage was not found in the color-location and shape-location tasks, suggesting that this effect does not simply reflect a cost of the number of objects to be compared with the memory representation. In the color-location and shape-location tasks, any cost of number of comparison stages might be reduced because filtering out the irrelevant features simplified the memory representation, producing a ceiling effect. To confirm that the lack of single-probe advantage and of interference from task irrelevant features in the color-location and shape-location tasks in Experiment 1B are not due to ceiling effects, Experiment 2 compared a feature switch detection task with a two-feature change task.

## Experiment 2: Control Experiment

In Experiment 2, we compared memory for binding information and memory for visual feature information to show that there is room for performance improvement on the color-location and shape-location binding tasks with a single probe. Recent studies [5], [9], [10] showed that change detection performance for feature conjunctions was worse than change detection for single features.

### Methods

#### Participants

Fourteen undergraduate students at Kyoto University participated in this study in exchange for class credit. All participants had normal color vision and normal or corrected-to-normal visual acuity. The procedures were approved by the internal review board of Graduate School of Human and Environmental Studies, Kyoto University, and written informed consent was obtained from all participants prior to the testing.

#### Materials and Procedure

**Figure 5:**
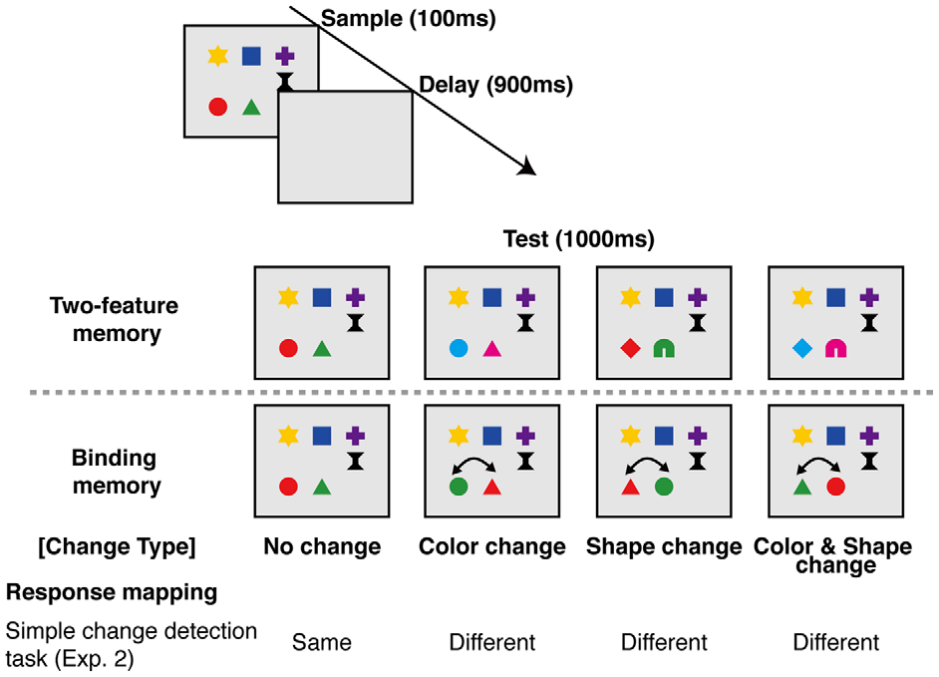
Schematic illustration of the control experiment. Four change types in the two-feature memory condition (no-change, color-change, shape-change, and location-change) and switch types in the binding-memory condition (no-switch, color-switch, shape-switch, and location-switch) and response mappings are illustrated.

Examples of the sample and test displays are illustrated in Figure 5. There were two memory conditions in this experiment: (1) a two-feature memory condition, in which features of two items could be replaced with new values not presented in the sample display, and (2) a binding-memory condition, in which features of two items could be swapped at test, as in the whole-probe condition in Experiment 1A and 1B. Three change types were used: color, shape, and color-and-shape. Note that the color-and-shape change in the binding-memory condition is identical to the location change condition in Experiment 1A and 1B. In the color-and-shape change trials in the two-feature memory condition, both color and shape were replaced with new values for the two objects. Thus, for each change type, the difference between the two-feature and the binding-memory conditions was that memory for feature-location binding was required for successful task performance in the binding-memory condition. In other words, the binding-memory condition required memory for color and location or shape and location, whereas the two-feature-memory condition merely required the list of colors or shapes. In this experiment, colors were selected from the following nine: red, yellow, green, blue, violet, white, orange, pink, and sky blue. Shapes were selected from the following nine: circle, triangle, square, cross, star, pillar, horseshoe, rhombus, and heart.

There were two experimental blocks of 120 trials for a total of 240 trials. Within each block, memory conditions (two-feature and binding) were randomized. Participants were given 24 practice trials before each experimental block.

### Results and Discussion

**Figure 6:**
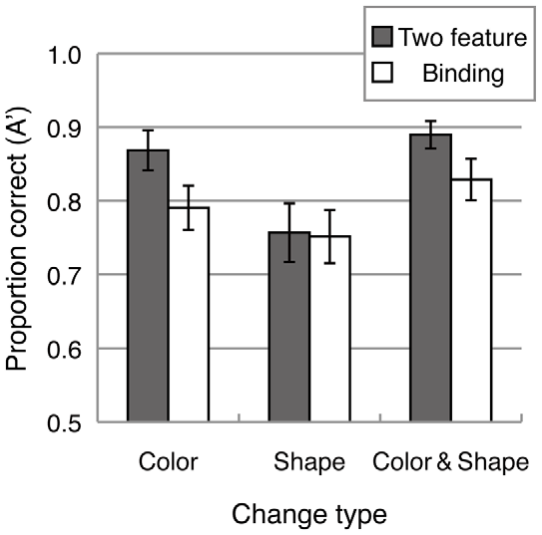
Mean sensitivity (A') for each change type and memory condition in Experiment 2.

Figure 6 shows mean A' for the two-feature memory condition and binding-memory condition. A 2 (memory condition: two-feature memory condition and binding-memory condition) ×3 (change type: color, shape and color-and-shape) ANOVA revealed significant main effects of memory condition (F(1, 13)  = 28.673, p<.0005), change type (F(2, 26)  = 31.106, p<.0001), and a significant interaction (F(2, 26)  = 4.440, p<.05). The analysis of the simple main effect revealed that task performance in the two-feature memory condition was significantly higher than in the binding-memory condition for color change (F(1, 39)  = 20.054, p<.0005) and color-and-shape change (F(1, 39)  = 12.258, p<.005), but not for shape change (F(1, 39)  = 0.060, n.s.). Overall performance in the binding-memory condition was fairly low (0.75∼0.8 in A'), and significantly lower than that in the two-feature memory condition with the color and color-and-shape change types, arguing against the possibility that the results in the color-location and shape-location conditions in Experiment 1B reflected ceiling effects.

One unexpected result was that there was no advantage for two-feature memory on shape change trials, but this was likely due to the difficulty of the two-feature memory condition. Increasing the number of shapes may have made even simple feature memory for shapes more difficult. Moreover, comparison of the whole-probe conditions in Experiment 1 and the binding-memory condition in Experiment 2 reveals that the relative difficulty across change type conditions was consistent across experiments, suggesting that differences in stimuli and tasks between Experiments 1 and 2 did not affect the relative difficulty of change types.

The significant improvement in performance in the two-feature memory condition implied that the results in the color-location and shape-location conditions in Experiment 1 do not reflect ceiling effects. However, because performance of the bind-memory condition in Experiment 2 was worse than those of the color change conditions of Experiments 1, there might be another explanation: the binding memory performance might get worse when the binding-memory condition was mixed with the two-feature memory condition. These interpretations depend on the difference between subject groups in Experiment 1 and 2. If the different performance between the bind-memory condition in Experiment 2 and the color change conditions in Experiment 1 was interpreted as the result of between-subject difference, the results of experiment 2 would indicate that the lack of the single probe advantage in the color-location and shape-location tasks in Experiment 1 does not reflect ceiling effect. In contrast, if considering that the subject groups in Experiment 1 and 2 were comparable, then the result of Experiment 2 would merely reflects the effect of task context in which the binding-memory condition was mixed with the two-feature memory condition. To examine these hypotheses, it is necessary to compare the performance in the bind-memory condition both with and without two-feature memory condition in a within-subject design in future research.

## General Discussion

In the present study, we examined selective encoding in VWM by systematically investigating interference from task-irrelevant features. Overall, encoding flexibility is feature-specific, which is consistent with the object file theory. Interference from the task-irrelevant feature was only observed in the color-shape condition, suggesting that color and shape information could be successfully filtered out, but location information could not. Therefore, although location information is added to object representations independent of task demands in a relatively automatic manner, other features (e.g., color, shape) can be flexibly added to object representations.

The second purpose of the current study was to examine the single-probe advantage, often referred to as an index of binding memory. We investigated the single-probe advantage when three features (color, shape and location) had to be bound (Experiment 1A), and in a relevant-feature switch detection task (Experiment 1B). In Experiment 1A, we found no evidence for the single-probe advantage, demonstrating that the single-probe advantage does not generalize to objects defined by a conjunction of three features. In Experiment 1B, the single-probe advantage was only significant in the color-shape condition. To confirm that the lack of single-probe advantage or interference in the color-location and shape-location conditions in Experiment 1B was not due to ceiling effects, Experiment 2 compared performance on a feature switch detection task with a feature change task, and showed that performance on the switch detection task was not at ceiling.

### Encoding Flexibility in VWM

The current study revealed that interference and a single-probe advantage only occurred in the color-shape condition, suggesting that location could not be filtered out, whereas color and shape could. This finding is largely consistent with the object file theory, but is inconsistent with the claim by Treisman and Zhang [6]. The apparent discrepancy between the current study and Treisman and Zhang [6] may be due to differences in methodology. The critical experiments for the automatic binding interpretation presented by Treisman and Zhang [6] showed that binding changes produced interference even when the items were presented at new locations in a feature change detection task. This location-independent interference from binding suggests that features are bound together even when feature binding is task-irrelevant. However, the feature change task used by Triesman and Zhang [6] contained both color and shape changes, so participants were required to encode both color and shape. In contrast, our relevant-feature switch detection task required ignoring the task-irrelevant feature entirely. Taken together, these results suggest that a necessary condition for successful flexible encoding may be that a feature as a whole is task-irrelevant at both an individual feature-level and a conjunction-level. Even if feature conjunctions are task-irrelevant, feature bindings are automatically encoded when both feature dimensions are task-relevant [6].

Logie et al. [11] recently examined the respective contributions of location, shape, and color to the formation of bindings of features in visual short-term memory (VSTM) across a range of study–test intervals. In their paradigm, one presented feature was designated as irrelevant and then randomized from study to test. They showed that, at shorter study–test intervals, randomizing location was most disruptive compared to shape and color. At longer intervals, randomizing any task-irrelevant feature had no impact on change detection for bindings between features, and location had no special role. Their results suggest that location is crucial at shorter study–test intervals but once representations are formed in VSTM, location loses that special status at longer intervals.

An important difference between the present study and earlier ones is that whereas the earlier studies mentioned above randomized presented feature designated as irrelevant from study to test, the present study did not. Taken together, our study was consistent with Logie et al. [11], which showed changes in location-feature binding led to significant interference at shorter study–test intervals, but was inconsistent with Triesman and Zhang [6]. On the other hand, including longer intervals, Logie et al. [11] showed randomizing any task-irrelevant feature had no impact on change detection for bindings between features, which was inconsistent with our study and Triesman and Zhang [6].

Although we interpreted interference or the lack thereof as reflecting VWM encoding rather than retrieval and/or matching, behavioral data in the current study cannot exclude the possibility that all object features are automatically encoded, but some object features (e.g., colors and shapes) can be successfully filtered out at the time of memory retrieval and matching. Some evidence for selective encoding and maintenance using ERPs may be beneficial for resolving this issue.

### Determinants of the Single-Probe Advantage

In the current study, a single-probe advantage was only observed in the color-shape condition, suggesting that the single-probe advantage mainly reflects interference from task-irrelevant features. However, the results are inconsistent with previous studies. Possible factors underlying these discrepancies are discussed below.

The significant single-probe advantage in the color-shape condition in the current study is consistent with Wheeler and Treisman [5], but inconsistent with Treisman and Zhang [6] and Johnson, Hollingworth, & Luck [12]. Treisman and Zhang attributed this discrepancy to differences in stimulus displays. In Wheeler and Treisman [5], objects in whole-probe test displays shared locations with objects in the sample display, but single-probe displays showed the target object in a new location. Therefore, the whole-probe cost may partly reflect an effect of overwriting. In Treisman and Zhang [6], half of the trials shared locations in both whole- and single-probe conditions, and a whole-probe advantage was observed, which may reflect a benefit of configurational cueing. The other half of the trials presented test objects in new locations, and the single-probe advantage was observed. However, this account cannot explain the current results, because the current study used shared locations in both whole- and single-probe conditions, suggesting that overwriting is not the critical factor for the single-probe advantage. Furthermore, Johnson et al. [12] failed to replicate Wheeler and Treisman [5] using nearly identical stimuli and experimental design.

One factor to be considered is that only Wheeler and Treisman [5] evaluated the single-probe advantage in a between-subjects design. Thus, it is possible that the main reason that Johnson et al. [12] failed to replicate their results is because they used a within-subjects design, which likely leads participants to prepare for both whole-probe and single-probe conditions. This task strategy leads to greater weighting of spatial configuration and less weighting of feature bindings than the single-probe condition in a between-subjects design, producing a whole-probe advantage rather than a single-probe advantage.

The next question, then, is why we found a significant single-probe advantage in the present study, despite the use of a within-subjects design. Our hypothesis is that the single-probe advantage is related to the effectiveness of binding object features to item locations. In the current study, the positions of sample display items were retained, with the exception of location-switch conditions, which may have encouraged position-based encoding to keep color-shape conjunctions. In contrast, the new position condition in Treisman and Zhang [6] and random location assignment in the probe display in Johnson et al. [12] may have discouraged participants from position-based encoding.

The lack of a significant single-probe advantage in the color-location condition in the current study is also inconsistent with Wheeler and Treisman [5]. Again this discrepancy probably reflects a difference between within- and between-subjects experimental designs. In the current study, we used a within-subjects design, where participants presumably employed a strategy to effectively deal with both whole- and single-probe displays, while Wheeler and Treisman [5] used a between-subjects design, where participants likely employed different optimal strategies for each probe condition.

Finally, the lack of a single-probe advantage in a simple change detection task with objects defined by a conjunction of three features (Experiment 1A) does not correspond to any previous studies, but may provide some clues for mechanisms underlying the single-probe advantage. The single-probe advantage observed in the within-subjects design may reflect a reduction of interference from task-irrelevant features. Thus, even with more complex conjunctions of three features, no single-probe advantage was obtained because no feature was task-irrelevant. This does not preclude the possibility that the single-probe advantage in the between-subjects design reflects feature binding in general, in which case the single-probe advantage may be observed in a simple change detection task with stimuli defined by conjunctions of three features. Further studies are necessary to resolve these issues.

### Relation to Other Selective Processing in VWM

Vogel, McCollough, & Machizawa [13] has shown that VWM capacity is closely related to an individual's ability to exclude irrelevant items from current tasks, an arguably important element of central executive control. While selective processing in their task is based on particular feature values (e.g., red, and upper visual field), selection in the current task is based on a whole feature dimension (color, shape, or location). One empirical question is whether the task-irrelevant, but selection-relevant, feature value can be filtered out. For example, in Vogel et al. 's task, participants are required to maintain orientation-location binding for red items while ignoring blue items. Thus, once irrelevant blue items are filtered out, color information becomes irrelevant to the change detection task. Whether color information can be filtered out even when it is necessary for initial selection will provide important information regarding mechanisms underlying selective encoding in VWM based on feature values.

Recently, a number of studies have shown that attentional selection of items in VWM is possible using location cues [14], [15] and feature cues [16]. These studies are currently limited to selection based on specific feature values. An interesting future study would be to examine whether dimension-based selection (or filtering) can be carried out with memory representations in VWM, which may substantially extend our understanding of flexibility of VWM.
